# Production and characterization of avian crypt-villus enteroids and the effect of chemicals

**DOI:** 10.1186/s12917-020-02397-1

**Published:** 2020-06-05

**Authors:** Mohan Acharya, Komala Arsi, Annie M. Donoghue, Rohana Liyanage, Narayan C. Rath

**Affiliations:** 1 Poultry Production and Product Safety Research Unit, ARS/USDA, Fayetteville, AR 72701 USA; 2 Department of Poultry Science, University of Arkansas, Fayetteville, AR 72701 USA; 3 Statewide Mass spectrometry Facility, Department of Chemistry and Biochemistry, University of Arkansas, Fayetteville, AR 72701 USA

**Keywords:** Avian intestinal mucosa, Enteroids, Immunochemical characterization, Effects of chemicals

## Abstract

**Background:**

Three-dimensional models of cell culture such as organoids and mini organs accord better advantage over regular cell culture because of their ability to simulate organ functions hence, used for disease modeling, metabolic research, and the development of therapeutics strategies. However, most advances in this area are limited to mammalian species with little progress in others such as poultry where it can be deployed to study problems of agricultural importance. In the course of enterocyte culture in chicken, we observed that intestinal mucosal villus-crypts self-repair and form spheroid-like structures which appear to be useful as ex vivo models to study enteric physiology and diseases.

**Results:**

The villus-crypts harvested from chicken intestinal mucosa were cultured to generate enteroids, purified by filtration then re cultured with different chemicals and growth factors to assess their response based on their morphological dispositions. Histochemical analyses using marker antibodies and probes showed the enteroids consisting different cell types such as epithelial, goblet, and enteroendocrine cells typical to villi and retain functional characteristics of intestinal mucosa.

**Conclusions:**

We present a simple procedure to generate avian crypt-villous enteroids containing different cell types. Because the absorptive cells are functionally positioned outwards, similar to the luminal enterocytes, the cells have better advantages to interact with the factors present in the culture medium. Thus, the enteroids have the potential to study the physiology, metabolism, and pathology of the intestinal villi and can be useful for preliminary screenings of the factors that may affect gut health in a cost-effective manner and reduce the use of live animals.

## Background

Organoids are three-dimensional assembly of cells capable of mimicking organ functions in culture hence, have been used for studies relating to regenerative medicine, metabolic research, disease modeling, and therapeutic developments [1–4]. Typically, the organoids are generated from primary tissues, progenitor stem cells, or induced pluripotent cells (IPSC), and maintained using a variety of growth and differentiation factors, and extracellular matrix supports [5]. The intestine is a vital organ for nutrient absorption and its epithelial linings protect the organism against harmful antigens, toxins, and pathogens that passage through it [6, 7]. Intestinal organoids, pioneered by studies of Sato et al. [8], have been used to understand their developmental physiology, culture of enteropathogens, and to study gastrointestinal problems, such as relating to epithelial integrity, enteritis, and autoimmunity [9–11]. However, most advances in this area have been limited to mammalian models largely, to mouse, human and to some extent, the porcine and bovine models of agricultural importance where the organoids are often generated using either progenitor stem cells in the crypts or induced pluripotent cells [12–17]. In avian species enteric organoid research is very limited although the poultry constitute a major human food source that are also vulnerable to different enteric problems that can affect their growth and susceptibility to diseases. The lack of pluripotent stem cells to generate intestinal organoids in avian species further limits the progress although there are some reports of success using intestinal crypt cells [18–20]. The poultry intestinal organoids can be useful as a screening tool to study the effect of nutrients to promote growth, understand the factors that can affect intestinal health, pathogen interactions, and screen for antibiotics alternatives to prevent zoonotic diseases. However, most current methods of generating intestinal organoids involve complex manipulations such as the use of different growth factors, extracellular matrix supports and inhibitors which often are mammalian specific and can be time consuming [21]. Further, in stem cell generated organoids, the villus growth are inwards that preclude the absorptive cells to align externally to the culture medium [22]. Large scale screening assays may be achievable if the organoids can be generated rapidly and cost-effectively. In the course of developing chicken enterocyte culture [23, 24], we observed that the villus crypts of intestinal mucosa tend to self-repair to form spheroid-like structures resembling mini organoids that appear to have potential to study villus physiology. Hence, the objective of this study was to streamline the procedure, purify avian villus enteroids, characterize them, and study their potential as a screening tool using some selective chemicals and growth factors.

## Results

### Generation of enteric organoids and immunofluorescence characterization

**Fig. 1:**
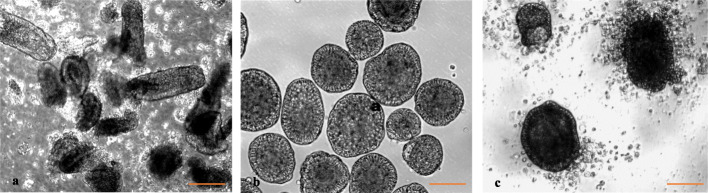
**a** Intestinal mucosa with villi following harvest, **b** purified enteroids after 24 h of culture, and (**c**) purified enteroids cultured additionally for 24 h showing extrusion and accumulation of cells around the enteroids. Magnification 200X. (Bar =100 μm)

Figures 1a and b show the intestinal mucosal villi following their harvest and after 24 h of culture when the enteroids were purified. Filtration process yielded substantially cleaner preparation of the enteroids, free of debris and adherent cells. The purified enteroids on further culture showed progressive accumulation of cells around them resulting from epithelial cell extrusion (Fig. 1c). The villus enteroids showed virtual peripheral lining of epithelial cells albeit they were spheroids and contained some core tissues often visible as orange tinge, likely due to some blood cells (not shown).

### Immunofluorescence staining of organoids

**Fig. 2:**
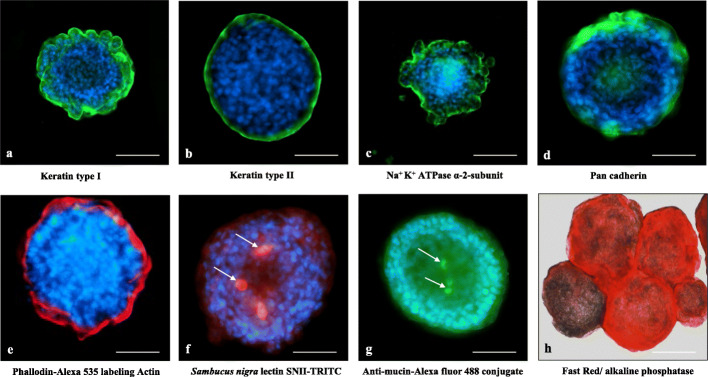
Immunolocalization of antigens in the villus enteroids: **a** and **b** keratin types I and II, **c** Na-K-ATPase α-2-subunit, **d** Pan cadherin, **e** actin binding alexa 535 labelled phalloidin, **f** goblet cells binding *Sambucus nigra* lectin SNII-TRITC, **g** goblet cells binding Anti-mucin antibody and (**h**) and Fast red positive alkaline phosphatase. The nuclei are stained blue with DAPI in all pictures. The magnification of the images in **a**, **c**, and **h** are 200 X, bar = 80 μm and the rest 400X, bar = 40 μm

**Fig. 3:**
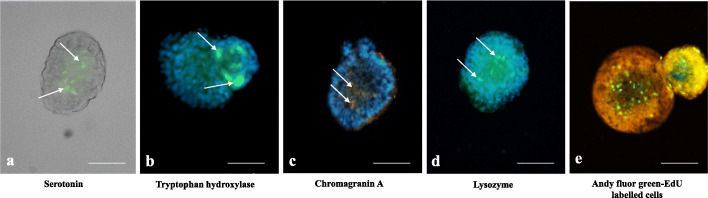
Immunofluorescence localization of antigen specific cells and proliferating cells. **a** faint green serotonin positive cells without counter stain, **b** tryptophan hydroxylase positive faint green cells, **c** chromogranin A positive cells identified in saffron color, **d** lysozyme positive cells, faint green, and (**e**) Andy fluor green fluorescent EdU labeled proliferating cells show as fluorescent green nuclei. The nuclei were stained blue with DAPI except in (**e**) where they were stained with propidium iodide showing orange-red color. Images are magnified to 200X, bar = 40 μm

Immunofluorescence localization of selective antigen markers associated with different cell types are shown in the Figs. 2a-h. The enteroids were positive for both keratins type I and II, Na-K-ATPase, pan cadherin, actin, mucin, and alkaline phosphatase. Keratins localized as peripheral bands in the enteroids (Figs. 2a, b). Cadherin, an adhesion molecule which occurs in the epithelium as e-cadherin, a tight junction associated protein [25, 26], was also present in the enteroids (Fig. 2d). Actin which occurs as a crescent band in the apical region of the epithelial enterocytes [24], showed strong presence (Fig. 2e). Goblet cells producing mucin were identified by their binding to *Sambucus nigra* lectin [24] and anti-mucin antibodies (Figs. 2f, g). The enteroids were positive for alkaline phosphatase identified by Fast red substrate (Fig. 2h). Some cells in the enteroids, that stained positive for serotonin, chromogranin A, and tryptophan hydroxylase, were presumed to be enterochromaffin cells (Figs. 3a-c), whereas those positive for lysozyme (Fig. 3d) were presumed to be cells producing antimicrobial factor such as the Paneth cells. Because of the spherical nature of the enteroids, it was not possible to ascertain whether these cells were crypt associated. Most of these cells other than the epithelial cells appeared as clusters or isolated populations of cells in the enteroids. The enteroids showed cell proliferation indicated by Andy fluor labeling of EdU positive cells which appeared bright green fluorescent, and scattered randomly over the organoids whereas the non-dividing cells appeared orange to red fluorescent (Fig. 3e).

### Alkaline phosphatase activity

Measurement of alkaline phosphatase activity with 4-nitrophenyl phosphate (4-NPP) substrate using 3 test chemicals showed no statistical difference with the control (Control: 3.21 ± 0.60, cGH: 2.40 ± 0.25; DSS: 2.65 ± 0.61; Serotonin: 2.71 ± 0.21 OD/μg protein, *n* = 3/group).

### The effect of different chemicals on organoid morphology

**Fig. 4:**
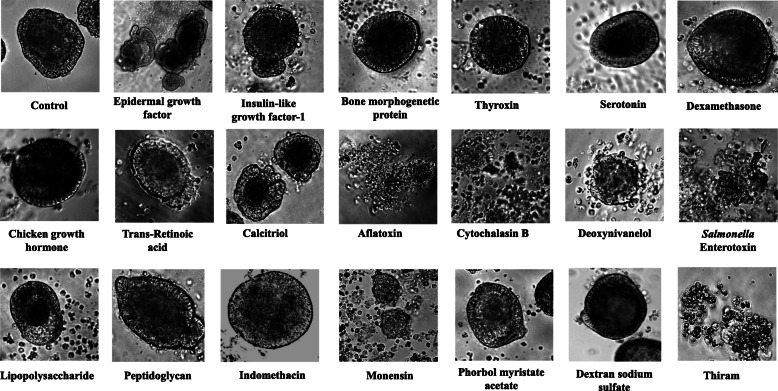
Shows the effect of different chemicals on the enteroids following 24 h of incubation with 1 μg/ml of each factor. Magnification 200X

Figure 4 and Table 1 show the representative images and the results of the effect of different chemicals on the enteroids after 24 h of treatment. Of the three tested growth factors only EGF and IGF-1 produced enterotrophic effects indicated by budding whereas the BMP-2 produced no significant effect at 24 h of incubation. In the hormone category, none of the tested hormones, except for dexamethasone showed any discernible effect. Dexamethasone appeared to shrink the epithelial cells. Of the two micronutrients tested, trans-retinoic acid showed no effect but 1, 25 dihydroxy vitamin D3 (calcitriol) appeared to shrink the core mass of the organoids and showed some budding activities. The mycotoxins adversely affected the enteroids causing their fragmentation and breakage by 24 h with deoxynivanelol (DON) showing some delayed effect compared with either aflatoxin B1 or cytochalasin B. The degenerative effect of DON was more pronounced at 48 h of incubation. Similar degenerative effects on the enteroids were evident by the treatment with both *Staphylococcus aureus* and *Clostridium perfringens* epsilon enterotoxins which not only caused vacuolation but also lead to the disintegration of the enteroids (not shown). *Salmonella typhimurium* lipopolysaccharide (LPS) appeared to produce some shrinkage of core tissues of the enteroids but the peptidoglycans produced no discernible changes up to 48 h of treatment. Indomethacin caused shrinkage of outer epithelial cells whereas the monensin caused degeneration of the enteroids. Phorbol myristate acetate (PMA) did not produce any discernible changes in the enteroids compared with the controls. Dextran sodium sulfate (DSS), produced no morphological changes whereas thiram, a fungicide, caused significant damage and degeneration of the enteroids (Fig. 4).

**Table 1:** Effect of chemicals on villus enteroids

**Category**	**Test chemicals**	**Supplier**	**Effect on enteroids**
***Growth factors***	Epidermal Growth factor (EGF)	Gibco, www.thermofisher.com	Supports budding, undulated periphery
	Recombinant human Insulin like growth factor (IGF1)	Gibco, www.thermofisher.com	Supports budding, undulation
	Bone morphogenetic protein-2 (BMP-2)	Peprotech, www.peprotech.com	No significant effect
***Hormones***	Thyroxin	Sigma, www.sigmaaldrich.com	No significant effect
	Dexamethasone	Sigma, www.sigmaaldrich.com	Epithelial cells impaired
	Chicken growth hormone (cGH)	Prospec (www.prospecbio.com)	No significant effect
	Serotonin	TCI, www.tcichemicals.com	No effect to some epithelial cell dystrophy
***Vitamins***	Trans-retinoic acid	Santa Cruz, www.scbt.com	No significant effect
	1, 25 dihydroxy vitamin D3 (calcitriol)	Biovision, www.biovision.com	Shrinking of central tissues
***Mycotoxins***	Aflatoxin B1	Cayman chemical, www.caymanchem.com	Enteroids degraded
	Deoxynivanelol (DON)	Cayman chemical, www.caymanchem.com	Enteroids degraded (48 h), delayed effect resulting in enteroid degeneration
	Cytochalasin B	Santa Cruz Biotechnology, www.scbt.com	Enteroids degraded
***Endo−/ Enterotoxins***	Lipopolysaccharide, (*Salmonella typhimurium)*	Santa Cruz Biotechnology, www.scbt.com	No significant effect
	Enterotoxin type B *(Staphylococcus aureus*)	List Biological, www.listlabs.com	Degradation of enteroids
	Epsilon enterotoxin *(Clostridium perfringens)*	List Biological, www.listlabs.com	Degradation of enteroids
	Peptidoglycan from *Saccharomyces cerevisiae*	Millipore Sigma, www.sigmaaldrich.com	No significant effect
***Metabolic modulators***	Indomethacin (prostaglandin inhibitor)	TCI chemicals, www.tcichemicals.com	Epithelial cells shrink
	Monensin (ionophore antibiotic, anticoccidial)	Sigma, www.sigmaaldrich.com	Degeneration of enteroids
	Phorbol myristate acetate (PMA) (protein kinase C activator)	AG Fluka, www.lab-honeywell.com	No significant effect
***Pesticide***	Tetramethyl thiuram disulfide (Thiram)	Sigma Chemicals, St. Louis, MO www.sigmaaldrich.com	Degeneration of enteroids
***Miscellaneous***	Dextran sodium sulfate (40 kDa)	Alfa Aesar, Canada	No significant effect

## Discussion

The villi are the major absorptive and protective units of intestine. Hence, understanding their physiology can provide insight into their role in regulating nutrient uptake, growth, and animal health problems. Taking advantage of the self-repairing ability of the villus crypts, we developed a simple method that uses standard cell culture medium that does not involve many special growth factors, conditioned media, and extracellular matrix gel supports to generate avian villus enteroids, that are often used to favor the clonal expansion of the crypt based progenitor cells. Each enteroid appeared to be a prototype unit of mucosal villus containing different cell types and showed cell turnover and shedding typical of the villi [27, 28]. The cell renewal, shedding and extrusions have been associated to active migration of cells along the villus which is associated with intestinal homeostasis [28]. Cell shedding was evident by the accumulation of a large number of free cells around the enteroids on subsequent days of culture. The enteroids also showed cell proliferation, evident from cell labelling studies and were indicative of their growth and budding activities although these activities tend to slow down. The presence of different cell types such as the epithelial, goblet, entero-endocrine cells, and the cells that produce antimicrobial peptides that are typical of villi [29, 30], and were evident from their respective markers. The antibodies against both keratins I and II, which bind to the epithelial cells [31, 32], were strongly reactive with the enteroids as was the antibody against Na-K-ATPase, an ion channel protein, responsible for the maintenance of intestinal health and the integrity of epithelial tight junction, cell motility and polarization [33, 34]. Decreased levels of Na-K-ATPase activities in the basolateral membrane of intestinal epithelial cells has been linked to chronic intestinal inflammation and malabsorption problems [35]. The enteroids also showed the presence of cadherin, a tight junction associated adhesion protein, that maintains epithelial barrier function [26, 36]. The mucin producing goblet cells were identified by their reactivity with SNII lectin [24, 37] and an anti mucin antibody. Of other specialized cells, the presence of enterochromaffin cells were indicated by the reactivity of the antibodies specific to serotonin, chicken tryptophan hydroxylase, and chicken chromagranin A which are the markers of these cells [38]. Lysozyme positive cells in the enteroids were indicated by their reactivity to an anti-lysozyme antibody. Paneth cells produce lysozyme but their presence in chickens has been controversial [39, 40] however, in a recent report, Wang et al. [41] showed the presence of these cells in the intestinal crypts. The location of lysozyme producing cells in the enteroids appeared to be surface associated. Many of the specialized cells such as goblet cells and enteroendocrine cells which occur in low numbers, appeared as scattered patches of cells. The enteroids were positive for alkaline phosphatase, an enzyme that is associated with enterocytes and implicated in the regulation of fatty acid absorption and protection of intestine against bacterial invasion [42]. Alkaline phosphatase also attenuates gut inflammation such as in colitis and is amenable to modulation by nutritional factors [43–45]. However, our results with 3 different factors, cGH, DSS, and serotonin, showed no significant modulation of alkaline phosphatase activity by any of these factors; nonetheless, the measurement of alkaline phosphatase can be a useful marker for enteroid function.

For its applications in poultry research, such as to understand the effect of nutrients, interactions with pathogens, or the screening of antibiotic alternatives, the villus organoids can be generated inexpensively and rapidly within days and serve as test models either as individual or collective units employing their morphological and biochemical changes. To test it, we investigated the effect of selective growth factors and chemicals that are known to affect intestine by assessing the gross changes in the morphologies of the enteroids. Our results showed both epidermal growth factor (EGF) and the insulin like growth factor-1 (IGF-1) to have trophic effect on the organoids. EGF is a major signaling protein implicated in intestine development and repair [46, 47] and insulin like growth factor-1 (IGF-1) causes intestinal proliferation [48], both of which promoted undulation and budding of the organoids within 24–48 h of treatment although the former was much more effective compared with the later. However, the bone morphogenetic protein-2 (BMP-2), another signaling protein and morphogen [49] that has been shown to affect intestinal epithelial differentiation [50], did not produce any significant enterotrophic effect. BMP’s effect on intestine has been suggested to be on the level of mesenchymal cells producing secretory cell differentiation [51]. Since, the enteroids have limited mesenchymal cells, the effect of BMP may not be morphologically discernible. Thyroxin, similarly, produced no significant morphological effect on the enteroids whereas dexamethasone produced some shrinking effect on epithelial cells without much effect on central cell mass. There are conflicting reports of the effect of thyroid hormones on intestinal epithelial cells with respect to growth although thyroxin has been shown to promote maturation of intestinal mucosa and help in calcium transport process [52]. Previously, using enterocyte culture, we observed the cells treated with thyroxin showed some morphological changes leaning towards more cuboidal shapes compared with the controls [24]. However, it was not apparent from the observation of the whole enteroids. The effect of dexamethasone, a synthetic glucocorticoid, to some extent, was consistent with the observations of Urayama et al. [53] who reported its shrinking effects on rat small intestinal cells. Serotonin, which is a product of enterochromaffin cells and other neural cells innervating gastrointestinal tract, are known to affect its motility, and possibly modulate Na+/K+ exchange system [54–56]. In this assay, serotonin produced little to no morphological changes in the organoids nor did it have any effect on their alkaline phosphatase activities that could be associated to Na-K-ATPase system. The chicken growth hormone (cGH) produced neither any morphological change nor affected alkaline phosphatase activity of the enteroids although GH is known to stimulate the growth of intestinal mucosa and proliferation of intestinal stem cells [57]. Trans-retinoic acid similarly, exhibited no morphological effect on the enteroids but the vitamin D3 (calcitriol) appeared to shrink the central mass of cells. Inhibitory and anti-proliferative effect of trans-retinoic acid has been reported in literature [58], but it was not evident from our results. The effect of vitamin D3 on proliferation or differentiation of enterocytes is little known although it plays a significant role in the absorption of calcium and phosphorous in the intestine [59]. Mycotoxins are fungal metabolites that are toxic to intestine [60]. Compared at the same concentration levels, both aflatoxin B1 and cytochalasin B caused lethal changes resulting in the fragmentation of enteroids within 24 h of treatment whereas deoxynivalenol (DON) appeared to be relatively less lethal and took longer incubation time, up to 48 h, to produce similar effects. The effects of mycotoxins are presumed to be due to their interfering effects on protein synthesis and cell division, and they disrupt intestinal cell barrier producing cellular apoptosis [61–63] which likely lead to their degeneration. Similarly, both *Staphylococcus aureus* and *Clostridium perfringens* epsilon enterotoxins were lethal to the enteroids and produced severe damage. Neither *Salmonella typhimurium* lipopolysaccharide nor fungal peptidoglycan produced any damaging effect on the organoids although both of these products are proinflammatory in many systems [64]. In a previous study with chicken enterocytes, LPS also did not produce any significant effect on the enterocytes [24], although, *Salmonella* infection has been reported to affect intestinal organoids causing their morphological changes and disrupting epithelial tight junctions [65]. It is not known whether the effects of *Salmonella* were due to their LPS content or due to the growth and invasion of the bacteria per se. The *Staphylococcus aureus enterotoxin* is well known for its wide range of damaging effects on intestine [66] and the enterotoxin of *Clostridium perfringens* has been identified as a major virulent factor that causes necrotic enteritis in livestock including poultry [67–70]. Besides, the *Clostridium difficile* toxin disrupts the epithelial barrier function of human intestinal organoids [71]. Both of the enterotoxins produced lethal effect on the enteroids.

With regards to metabolic modulators, the effect of indomethacin, a prostaglandin inhibitor, PMA, a protein kinase C activator, and monensin, an ionophore and anticoccidial antibiotic, on the enteroid morphologies were tested. Indomethacin produces ulcers in human and rat small intestines [72] although it may be a long term effect of the drug. In the current study, indomethacin appeared to only affect the outer epithelial cells of the enteroids without causing any lethality. Similarly, in a previous study using PMA where we observed significant cachectic effect of it on the enterocytes at concentrations of 1 μg/ml or less [23, 24], showed no significant effect on the enteroids. Monensin, an anticoccidial drug used in poultry production caused significant damage to the organoids and in some early studies it was reported to have toxic and necrotic effect on intestine [73]. Tetramethyl thiuram disulfide (thiram) is a fungicide and an endocrine disruptor, produces many toxic effects in chickens including gastrointestinal problems and growth retardation [74]. Thiram exhibited severe toxic effect on the enteroids leading to their disintegration but dextran sodium sulfate (DSS), a widely used inducer of experimental colitis, showed neither any damaging effect on the enteroids nor caused any change in their alkaline phosphatase activities. However, the in vivo effect of DSS is attributed to the changes in the mucosal permeability and the activation of inflammatory cells [75–77].

## Conclusion

The major highlight of this study is the method to generate chicken crypto-villus enteroids from mucosal tissues in a relatively simple and cost-effective manner precluding the use of complex growth factors and extracellular matrix supports. The structural mimicry of the enteroids to the villi can be useful for many routine in vitro assays and to study their physiology and their interactions with nutrients, chemicals, pathogens, and enteric disease problems. The advantage offered by the enteroids also stems from their diverse cell types and the positioning of the absorptive cells that directly expose to the culture medium as they would naturally in the lumen of the intestine, thus, making them amenable to different metabolic, developmental, genetic, and pathological studies. However, maintaining the enteroids for long term use may require additional maneuvers and, use of different growth factors and inhibitors that may prevent their collapse, enable their preservation, storage, and retrieval. Nonetheless, the villus enteroid model appears promising for its use in poultry research for routine screening assays that can also reduce the use of live animals.

## Methods

### Harvest of intestinal villi and culture to generate enteroids

**Fig. 5:**
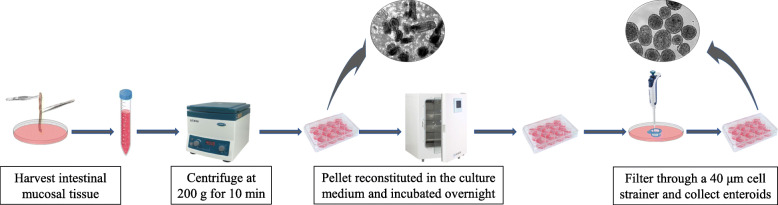
An illustration of the procedure for generating enteroids from intestinal villi

All animal procedures were approved by the Institutional Animal Care and Use Committee of the University of Arkansas. Day-old male broiler chicks (Cobb 500), a by-product from the female line, obtained from a local hatchery were euthanized by cervical dislocation, the intestine segments between the pancreatic loop and ileocecal junction were aseptically removed and placed in Dulbecco’s modified minimum essential medium containing Ham’s F12 nutrients (DMEM-F12,1:1) with glutamine, HEPES, sodium bicarbonate (HiMedia Laboratories, LLC), sodium pyruvate, and antibiotic-antimycotic supplements (Sigma Aldrich), referred to as ‘complete medium’. The mucosal tissues were extruded into fresh complete medium by draining intestine segments of 5–6 chicks longitudinally with the help of tweezers. The pooled tissues were dispersed by trituration and centrifuged at 200 *g* for 10 min to remove the supernatant. This process was repeated once after which the tissue pellets were re suspended in the above medium additionally supplemented with 10% Hyclone fetal bovine serum (FBS) (GE health Sciences, Logan UT), 1X insulin transferrin selenite (ITS), 1X polyamine (www.sigmaaldrich.com), and 1X bovine pituitary extract (Cell Applications Inc., San Diego, CA) as per the manufacturer’s recommendation, henceforth referred to as “culture medium’, for overnight incubation in hydrophobic suspension cell culture plates (Sarstedt, Germany). This process results in the sheared ends of the villi to self-repair and form spherical enteroids. The cultures containing cell debris and the enteroids were gently triturated to remove the attached cells then strained through 40 μm Falcon cell strainers (www.vwr.com) by three successive transfers and washes with excess volumes of complete medium. The enteroids were transferred from the strainer set in fresh medium using a pipette and concentrated by centrifugation at 200 *g* for 10 min, reconstituted in fresh culture medium containing FBS and growth factors for different assays (Fig. 5). The enteroids were cultured in hydrophobic suspension culture wells. The villus crypts and the enteroids were photographed using a BX Olympus inverted microscope equipped with a CoolSnapPro camera (www.mediacy.com).

### Immunofluorescence staining of enteroids and cell proliferation assay

The presence of different marker antigens in the enteroids were assessed by immunofluorescence localization using chicken antigen specific antibodies and other probes. The enteroids at 48 h of culture were transferred to glass slides coated with 2% Biobond™ (Electronmicroscopy Science, www.emsdiasum.com) using glass capillaries, fixed with 4% *para*-formaldehyde in phosphate buffered saline (PBS) for 15 min in vapor phase, and stored for subsequent histochemical procedures. For antigen localization, the slides were washed twice 5 min each in excess PBS then the enteroids were permeabilized with 0.5% Triton X-100 in PBS, and blocked with 10% goat serum for 10 min. Primary antibodies (Table S1) diluted per manufacturer suggested concentrations where available, or diluted to an approximate concentration of 5 μg/ml in 0.1% bovine serum albumin (BSA) for monoclonal antibodies, were placed on the enteroids, covered with a parafilm, and incubated overnight at 4 °C in a humidified chamber. The enteroids were washed 3 times with PBS for 5 min each and layered over with secondary antibodies such as goat anti-mouse IgG or goat anti-rabbit IgG conjugated to Alexa fluor 488 (Abcam, Inc) using the manufacturer suggested concentrations as probes against their respective primary antibodies, incubated for 1 h at room temperature, and rinsed 3 times with PBS. Appropriate negative control tests were done using only the secondary antibodies and omitting the primary antibodies. In preliminary screenings some of the primary antibodies that were not chicken specific, showed no specific binding. Following incubation with the antibodies, the enteroids were counterstained with 4′, 6-diamidine-2′-phenylindole dihydrochloride (DAPI; 0.2 μg/ml PBS), and applied Cytovista™ tissue cleaning agent (www.thermofisher.com) for 10 min, washed twice with excess PBS, and mounted with ProLong gold anti-fade reagent (www.thermofisher.com). Actin was stained using Alexa fluor 535 labelled Actistain Phalloidin (www.cytoskeleton.com) following the manufacturer’s protocol. Tetramethyl rhodamine (TRITC) conjugated *Sambucus nigra* lectin (SNAII, EY laboratories Inc., San Mateo, CA) was used to identify mucin producing cells [24, 37]. The fluorescent and bright field images were photographed with an Olympus BX microscope equipped with Infinity 3-3UR camera (www.lumenera.com). The images were processed and superposed using proPremier software (www.mediacy.com/imagepro). In some instances, the labelled target cells were pseudo colorized to enhance contrast and visibility of the antigen positive cells. The antibodies and the probe reagents are listed in the supplemental information (Table S1).

The proliferation of the cells in the enteroids was monitored using iClick™ EdU Andy Fluor 488 imaging kit (ABP Biosciences, www.abpbio.com). The enteric spheroids were plated in 48 well plates and incubated overnight with 10 μM 5-ethynyl-2′-deoxyuridine (EdU). Twenty-four hours later, the enteroids were washed 2 times in serum free DMEM/ F-12 medium and transferred to slides prepared as described above, and fixed with 4% *para*-formaldehyde. The enteroids were permeabilized with 0.5% triton X-100, washed 3 times in PBS, layered over with ~ 0.5 ml of iClick reaction cocktail as suggested by the manufacturer, and incubated for 30 min at room temperature in dark. The enteroids were then counter stained with propidium iodide (1 μg/ml PBS) as nuclear stain, and the fluorescent images visualized under microscope. The proliferating cells were bright green fluorescent whereas the non-proliferating cells appeared orange to red.

### Alkaline phosphatase immunohistochemistry and assay

Intestine is a major source of alkaline phosphatase which helps in the absorption of fatty acids and is an important protective enzyme against inflammation [78, 79]. We used a Fast-Red chromogen kit (www.Abcam.com) for histochemical identification of alkaline phosphatase in the enteroids. Quantitative measurement of the alkaline phosphatase activity was done using 4-nitrophenyl phosphate (4-NPP) substrate in diethanolamine buffer pH 9.7. The effect of cGH, DSS, and serotonin on enteroid alkaline phosphatase activities were measured incubating ~ 15–20 enteroids in 100 μl of culture medium in suspension culture plates (Sarstedt, Germany). The test chemicals were added at the concentrations of 1 μg/ml for 24 h then transferred to microtubes and centrifuged at 1000 *g* to remove supernatant. The enteroid pellets were additionally washed in 0.5 ml of Hanks Balanced salt solution (HBSS), centrifuged at 10,000 *g* and the resulting pellet dissolved in 30 μl of *M-Per* lysis buffer (Thermo Fisher Scientific). The lysates were centrifuged at 20,000 *g* and the alkaline phosphatase activity of the supernatants measured incubating an aliquot of the supernatant with 100 μl of substrate solution prepared dissolving 4-NPP (1 mg/ml) and incubating at 37 °C for 30 min when the reaction was terminated adding an equal volume of 1 N NaOH [80]. The optical density (OD) of 4-nitrophenol was determined at 405 nm. The protein concentrations of the extracts were determined using BCA reagent (Thermo Fisher Scientific) using bovine serum albumin (BSA) as standard. Alkaline phosphatase activity was calculated against the protein content of the extract. The assays were done using triplicate cultures, results evaluated statistically using GLM procedure [81] and expressed as OD / μg protein at 30 min of incubation at 37 °C.

### The effect of different factors on enteroids

To find the effect of different chemicals on the enteroids, we used a panel of selective growth factors, hormones, micronutrients, toxins, and chemicals (Table 1). Approximately 7–10 enteroids were placed in 100 μl culture medium in 96 well suspension culture plates and treated with the chemicals for 24–48 h and the overall changes in the enteroids were assessed under microscope. The test chemicals were first dissolved in their respective solvents such as complete medium (growth factors), or ethanol (for hydrophobic chemicals) as recommended by the manufacturer then diluted further with complete medium such as the final concentration of ethanol not to exceed over 1% in culture.. For serotonin we used ascorbic acid (20 mg/ml) as diluent and antioxidant. The comparisons were done utilizing only a single concentration of all chemicals and growth factors, 1 μg/ml although preliminary tests were run using log dilutions of some of the chemicals. Solvents diluted appropriately were used as controls and each assay was done in triplicate and repeated twice at 2 different times. Enteroids were observed for any significant changes in their morphologies and photographed using a BX Olympus microscope as described above. The subjective but independent assessments of the morphological changes were done by 2 individuals. The overall effects on the villus enteroids are shown in Fig. 4 and Table 1.

## Supplementary information

**Additional file 1: Table S1.** Antibodies, probes, and their targets.

## Data Availability

The datasets used and/or analyzed during current study are available from the corresponding author on reasonable request.

## Abbreviations

BCA: Bicinchoninic acid
BMP-2: Bone morphogenetic protein-2
cGH: Chicken growth hormone
DAPI: 4′, 6-diamidine-2′-phenylindole dihydrochloride
DON: Deoxynivanelol
DSS: Dextran sulfate sodium
EGF: Epidermal growth factor
IGF-1: Insulin like growth factor 1
LPS: Lipopolysachharide
PBS: Phosphate buffered saline
PMA: Phorbol myristate acetate
SNII: *Sambucus nigra* lectin II
