# Supplementary material for: Production and characterization of avian crypt-villus enteroids and the effect of chemicals
Source: BMC Vet Res. 2020 Jun 5;16:179. doi: 10.1186/s12917-020-02397-1 (PMC7275437; doi:10.1186/s12917-020-02397-1)
Supplement: Supplementary file 1 — Additional file 1: Table S1. Antibodies, probes, and their targets. [file 12917_2020_2397_MOESM1_ESM.docx]

**Table S1.** Antibodies, probes, and their targets

| **Antibodies or Probes** | **Supplier** | **Target antigen** |
| --- | --- | --- |
| Anti-keratin type I  (monoclonal) | Millipore Sigma, [www.emdmillipore.com](http://www.emdmillipore.com) | Keratin (epithelial cells) |
| Anti-keratin type II  (mouse monoclonal) | Millipore Sigma, [www.emdmillipore.com](http://www.emdmillipore.com) | Keratin (epithelial cells) |
| **α-2 F-S** (chicken Na-K-ATPase α subunit) (Mouse monoclonal) | Developmental Studies Hybridoma Bank (DSHB), University of Iowa, [www.dshb.biology.uiowa.edu](http://www.dshb.biology.uiowa.edu) | Na-K-ATPase, (ion channel protein) |
| Anti-chicken pan cadherin (Rabbit polyclonal) | Abcam, [www.abcam.com](http://www.abcam.com) | Epithelial cell cadherin (adhesion molecule) |
| Phalloidin-Alexa fluor 535 | Cytoskeleton Inc., [www.cytoskeleton.com](http://www.cytoskeleton.com) | F-actin |
| *Sambucus nigra* lectin SNII-TRITC | EYlabs, [www.EYlabs.com](http://www.EYlabs.com) | Mucin (goblet cells) |
| Anti-mucin-alexa fluor 488 conjugate (monoclonal) | Santa Cruz BT, [www.scbt.com](http://www.scbt.com) | Mucin (goblet cells) |
| Fast-Red substrate kit | Abcam, [www.abcam.com](http://www.abcam.com) | Alkaline phosphatase |
| Anti- serotonin (Rabbit polyclonal) | Enzo Life Sciences, [www.enzolifesciences.com](http://www.enzolifesciences.com) | Enterochromaffin cells |
| Anti-tryptophan hydroxylase (monoclonal) | Immunostar Inc., [www.immunostar.com](http://www.immunostar.com) | Enzyme involved in the synthesis of serotonin (Enterochromafin cells) |
| Anti-chromogranin A (Rabbit polyclonal) | Immunostar Inc., [www.immunostar.com](http://www.immunostar.com) | Enterochromaffin cells |
| Anti-chicken lysozyme | Enzo Life Sciences, [www.enzolifesciences.com](http://www.enzolifesciences.com) | Paneth cells? |
| iClick^TM^ EdU Andy Fluor 488 imaging kit | (ABP Biosciences, [www.abpbio.com](http://www.abpbio.com)) | Proliferating cells |
